# Chemical Characterization of Dew Water Collected in Different Geographic Regions of Poland

**DOI:** 10.3390/s8064006

**Published:** 2008-06-25

**Authors:** Żaneta Polkowska, Marek Błaś, Kamila Klimaszewska, Mieczysław Sobik, Stanisław Małek, Jacek Namieśnik

**Affiliations:** 1 Department of Analytical Chemistry, Chemical Faculty, Gdansk University of Technology (GUT), 11/12 G. Narutowicza St., 80-952 Gdańsk, Poland; E-Mails: skakama@chem.pg.gda.pl; chemanal@pg.gda.pl; 2 Department of Meteorology and Climatology, Institute of Geography and Regional Development, University of Wrocław, 8 Kosiby Street, PL-51670, Wrocław, Poland; E-mails: blasm@meteo.uni.wroc.pl; sobik@meteo.uni.wroc.pl; 3 Forest Faculty, Agricultural University of Cracow, 29 Listopada 46, 31-425 Cracow, Poland.

**Keywords:** Urban Environmental Monitoring, Dew, Ionic concentrations, Phenols, Formaldehyde, Ionic Correlations, Type of Air Mass, Synoptic Situation, Poland

## Abstract

The results of a dew monitoring program performed in Poland with the aim to outline the chemical composition of dew water in meteorological context are presented. Dew samples were collected from eight measurement stations from August 2004 to November 2006. Taking into account the type of land use and characteristics of pollutant emission, sampling sites were divided into the following categories: rural, coastal urban and inland urban stations. Selected anions and cations as well as formaldehyde and sum of phenols were determined. The average TIC (Total Inorganic Ionic Content) values in dew samples ranged from 0.83 to 3.93 between individual stations with 10.9 meq/L as the highest daily value of TIC measured. The average TIC values observed in dew at all stations were at a similar level (2.46 meq/L) when compared with hoarfrost (2.86 meq/L). However, these values were much higher in comparison with other kinds of atmospheric water like precipitation (wet only; 0.37 meq/L) or fog/cloud (1.01 meq/L). The pH values of dew water ranged from 5.22 to 7.35 for urban coastal stations, from 5.67 to 8.02 for urban inland stations and from 4.16 to 8.76 for dew samples collected in the rural area. HCHO was found in 97 % of dew samples, with concentrations ranging from 0.010 to 5.40 meq/L. Excluding stations near the seashore, where the contribution of Na^+^ and Cl^-^ increased, the most important ions were sulphates. A very low contribution of NO_3_^-^ and noticeable increase of Ca^2+^ which were not observed in the case of precipitation and fog water, were typical in all stations. The contribution of ammonium ion was two times higher at rural stations as a result of agricultural ammonia emissions. The strongest correlations were noticed between the sum of acidifying anions SO_4_^2-^ + NO_3_^-^ and Ca^2+^ ion for all urban and rural stations. A very strong correlation was also observed for Na^+^ and Cl^-^ ions in urban coastal stations, as a natural consequence of the location of these stations close to the sea. It was proved that thermal stratification, direction of circulation and local breeze circulation control the atmospheric chemistry at ground level, where dew is formed. The highest TIC values at urban stations were associated with anticyclonic weather, while at rural sites with cyclonic weather situations. The chemistry of dew water in urban coastal stations was closely related to local breeze circulation in the warm season, mainly in the form of diurnal breeze causing a significant increase of the concentration of Na^+^ and Cl^-^ions. Thus, dew can be a good indicator of the atmospheric pollution level at a given site. Taking into account both high TIC values and the annual water equivalent estimated at around 50 mm, dew is a considerable factor of wet deposition, responsible for an additional 60 % of pollutant input into the ground when compared with precipitation.

## 1. Introduction

Dew is the product of direct condensation of atmospheric water vapour on the ground, the temperature of which has fallen below the dew point but not as low as water is freezing point. The conditions leading to dew formation are 1) a radiating surface, well insulated from the heat supply of the soil, on which vapor may condense; 2) a clear, still atmosphere with low specific humidity in all but the surface layers, to permit sufficient effective terrestrial radiation to cool the surface; and 3) high relative humidity in surface air layers, or an adjacent source of moisture such as a lake [1]. An examination of dew formation on a microscale shows that different processes are involved in the growth of dew drops: direct accommodation at the drop surface in areas of maximum temperature gradients, nucleation and evaporation of clusters of near critical radius and coalescence of airborne small droplets formed earlier through nucleation [2].

In a major part of Poland dew deposition is the second most important path of water flux from the atmosphere to the ground after precipitation. In terms of frequency and duration in the warm-half of the year, dew is observed almost every night with scarce or scattered cloudiness and lasts even longer than precipitation. The annual number of days with dew in the lowland part of Poland varies from around 100 to 160, and it decreases on convex landforms to around 10-30 days in the case of conspicuous mountain summits and ridges above 1000 m a.s.l. The volume of annual dew deposition at different locations in Poland is disputable. Olszewski [3] measured it as around 6 mm with the use of a dew-recorder, whilst Hutorowicz's [4] measurements over 10 years revealed that dew formation was observed during 122 nights annually, with a total volume of 53 mm per year. The latter value is equal to around 10 % of annual precipitation. It could be also calculated as 0.4 mm per night, as a mean dewfall rate which corresponds to results presented in other studies [5-7].

Dew is a relatively neglected topic, even through measuring dew formation, the rate of accumulation and total deposition present interesting research questions [8, 9]. A major limitation in assessing the ecological and environmental role of dew is the extreme difficulty of collecting accurate measurements. In the majority of cases, dew is an important moisture input and plays a significant ecological function in most desert ecosystems [10]. In the NW Negev desert, long-term observations by Evenari *et al.* [11] show that dew occurs about 200 day per year and can reach the equivalent of 30 mm of annual precipitation. In drought years, dewfall can exceed annual rainfall. Interest also exists in urban dew and its implications for an urban climate and air pollution deposition [12, 13]. Measurements conducted in Vancouver showed that an asphalt shingle roof is one of the most efficient surfaces of dew accumulation [12]. Information regarding the chemical composition of dew is not often interpreted with meteorological data. Very limited studies have been done on dew chemistry worldwide [14-19].

While precipitation chemistry in Poland is described quite well, very limited information concerning the composition of dew exists. The aim of the present research was to present information about the chemical composition of dew water collected in different places (rural and urban) and compare these results with meteorological data (horizontal pressure gradient-hPa/111 km, synoptic scale atmospheric circulation, direction of circulation).

## 2. Experimental Section

**Figure 1.**
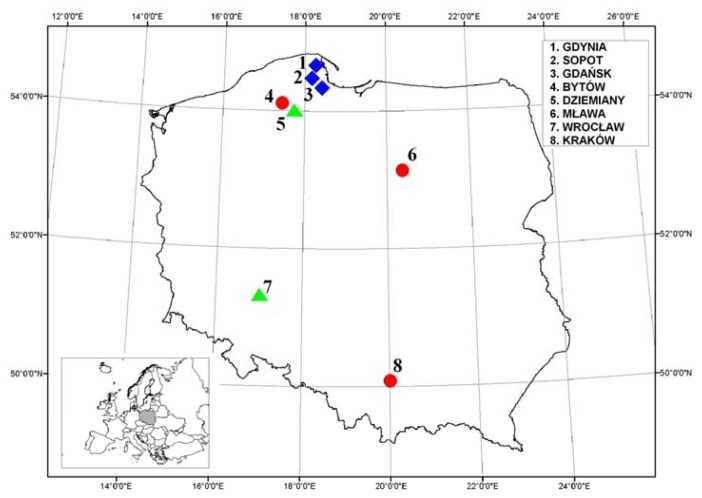
Locations of the sampling sites classified on the basis of landuse; coastal urban (1, 2 and 3), urban inland (4, 6 and 8) and rural stations (5 and 7).

Dew samples were collected from eight measurement stations from August 2004 to November 2006. Taking into account the type of land use and chatacteristics of pollutant emissions, the sampling sites were divided into three groups: rural stations (located in agricultural areas: Wrocław, Dziemiany); coastal urban stations (close to the coast line: Gdańsk, Sopot, Gdynia) and inland urban stations (the chemical composition of precipitation in a town, with increasing traffic intensity and industry: Bytów, Mława, Kraków). Table 1 summarizes the basic characteristics of the sampling sites and Figure 1 illustrates their geographical locations. Dew samples were collected using a sampler based on the design described by Muselli *et al.* [16], placed 50 cm above ground. Basic information concerning the applied sampler is presented in Table 2.

Droplets remaining on the collection surface were transferred to the collector using a polyethylene scraper. The dew samples were collected early in the morning. Before the expected appearance of dew, the collection surface was flushed with deionized water and subsequently dried. The collection of dew samples took place only on rainless nights to eliminate the influence of precipitation.

Samples were collected during or immediately after a deposition event. They were stored at a low temperature without chemical preservatives because the analysis was performed either directly on-site, or immediately after in the laboratory. Samples were analyzed for pH, volume, and conductivity. Select anions and cations were then quantified against a synthetic rain standard using ion suppressed chromatography (Dionex Corporation, USA). This synthetic standard is Reference Material No. 409 (BCR-409, Institute for Reference Materials and Measurements, Belgium) and Analytical Reference Material Rain-97 (National Water Research Institute, Environment Canada) [20]. Formaldehyde was determined spectrophotometrically (Merck, Germany) based on its reaction with chromotropic acid. In a solution acidified with sulphuric acid, formaldehyde reacted with chromotropic acid to form a violet dye that was measured [21]. In a buffered solution, in the presence of an oxidizing agent, phenol and its ortho- and meta-substituted compounds react with 4-aminoantipyrine to form a red compound that is determined spectrophotometrically. Instruments used for these measurements, listed in Table 3, were selected to assure reliable and reproducible results.

Date quality assurance was performed by evaluating the percentage difference of the ionic balance (PDI) and the sum of the total inorganic ionic content (TIC). PDI was calculated as:

$$\text{PDI}=\frac{\text{Conc}._{\text{anions}}-\text{Conc}._{\text{cations}}}{\text{Conc}._{\text{anions}}+\text{Conc}._{\text{cations}}}x100\;[22].$$

The acceptability criterion was set as PDI ≤ ± 20 % [23]. TIC represents the sum of liquid phase concentration of SO_4_^2-^, NO_3_^-^, Cl^-^, H^+^, NH_4_^+^, Ca^2+^, Mg^2+^, Na^+^ and K^+^[ 24]. The level of non-sea-salt sulfate (nss SO_4_^2-^) and non-sea-salt calcium (nss Ca^2+^) was estimated using the following equations:

$$\begin{array}{c} \text{nss}\;{{\text{SO}}_{4}}^{2‐}={{\text{SO}}_{4}}^{2‐}‐{\left({{\text{SO}}_{4}}^{2‐}/{\text{Na}}^{+}\right)}_{\text{seawater}}\cdot {\text{Na}}^{+}\\ \text{nss}\;{\text{Ca}}^{2+}={\text{Ca}}^{2+}‐{\left({\text{Ca}}^{2+}/{\text{Na}}^{+}\right)}_{\text{seawater}}\cdot {\text{Na}}^{+} \end{array}$$

where (SO_4_^2-^/Na^+^)_seawater_ and (Ca^2+^/Na^+^)_seawater_ are the concentration ratio of SO_4_^2-^ to Na^+^ and that of Ca^2+^ to Na^+^ in seawater, which are 0.12 and 0.044 (equivalent ratio), respectively [25].

In this paper, we use the acidifying potential:

$$\left(\text{AP}=[\text{nss}\;{{\text{SO}}_{4}}^{2‐}]+[{{\text{NO}}_{3}}^{‐}]\right)$$

and the neutralizing potential:

$$\left(\text{NP}=[{{\text{NH}}_{4}}^{+}{]+[\text{nss Ca}}^{2+}]\right)$$

to discuss both components. In order to ascertain the pH value, we use the pAi:

$$(\text{pAi}=‐\log([\text{nss}\;{{\text{SO}}_{4}}^{2‐}]+[{{\text{NO}}_{3}}^{‐}])\;[26].$$

The level of magnesium loss (loss Mg^2+^) was estimated using the following equation:

$$\text{loss}\;{\text{Mg}}^{2+}={\left({\text{Mg}}^{2+}/{\text{Na}}^{+}\right)}_{\text{seawater}}\cdot {\text{Na}}^{+}{‐\;\text{Mg}}^{2+}$$

where (Mg^2+^/ Na^+^)_seawater_ is the concentration ratio of Mg^2+^ to Na^+^ in seawater, which is 0.23 (equivalent ratio) [27].

## 3. Results and Discussion

### 3.1. Chemical composition of dew

The average TIC concentration (arithmetic, not volume weighted) in dew samples ranged from 0.83 in Wrocław to 3.93 in Kraków, where the highest daily value of TIC (10.9 meq/L) was measured. Such high TIC values were also observed at the urban inland station in Bytów (10.1 meq/L) and the urban coastal station in Gdynia (8.24 meq/L). Similar results for TIC were determined in dew samples collected in Amman (Jordan) – the maximum value of TIC was 7.68 meq/L [19], and in Bordeaux (France), the maximum value of TIC was 4.18 meq/L [15]. However, average TIC values in rural inland stations were around 50 % lower than in urban areas. The highest value was observed for Dziemiany (6.20 meq/L). The average TIC values observed in dew at all stations were at the same level (2.46 meq/L) when compared with hoarfrost (2.86 meq/L). However, these values were much lower in precipitation (wet only; 0.37 meq/L) and orographic fog water (1.01 meq/L) [28,29].

**Figure 3.**
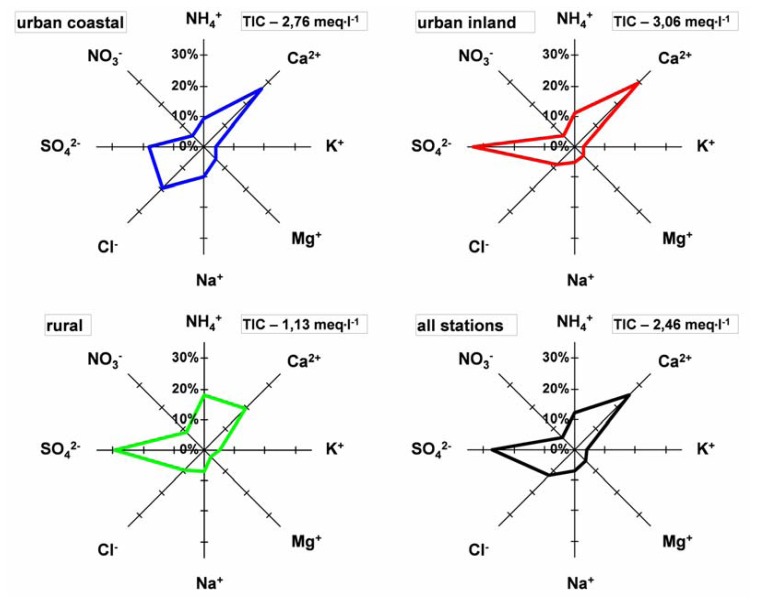
Chemical composition of dew water for different types of landuse in Poland: A-all stations; B-rural inland stations; C-urban coastal stations; D-urban inland stations.

The average Σ anions/Σ cations ratio for dew samples collected from different forms of land-use in Poland was calculated to be 0.92, whereas in Bordeaux (France), it was 1.03 [14]. More information concerning chemical composition, select hydrochemical indices and the percentage contribution of select ions determined in dew samples are reported in Table 4 and Figure 3.

Excluding stations near the seashore, the most important ions are sulphates. This concerns densely urbanized areas as well as rural ones. A noticeably smaller contribution of SO_4_^2-^ in dew collected at coastal urban stations can be related to the narrowing influx zone of these pollutants to the south, west and north-west (S-W-NW) sectors, due to limited inflow from the open sea side, i.e. at least from the whole eastern sector (E). However, the values of the nss SO_4_^2-^/SO_4_^2-^ ratio were close to 1 for all stations, which proved the anthropogenic origin of sulfate ions in dew samples.

For all of the dew water which was collected, NO_3_^-^ characteristically constituted only a small fraction of the ions. In comparison with precipitation and fog water, his was true regardless of the environment of the measuring stations. It seems that this results from the fact that dew is formed during the night when traffic intensity is much lower than during daytime (2.3 times lower). As a result of very fast NO_x_ conversion, the absence of its constant influx during the night may lead to its smaller concentration in dew samples. In agricultural areas with less urban influences, the contribution of ammonium ion was two times higher. This can be related to the higher influence of agricultural activity, which leads to considerably higher ammonia emission, very low emission and a very limited range of spatial deposition. In dew samples collected at coastal urban stations the contribution of ions of sea origin (Na^+^ and Cl^-^) increased. This results from the localization of these stations near the seashore. Regardless of the environment of the measuring stations, the contribution of Ca^2+^ ions noticeably increased (from 20 to 30 %). This contribution was much higher in dew in comparison with other kinds of atmospheric water (precipitation, fog). For frost, which can be regarded as a counterpart of mist at a temperature below zero, the contribution of Ca^2+^ in the total content of ions was two times lover. It seems that the main source of calcium is airborne dust from building areas, worn away concrete elements (curbs, pavements, roadways, etc.) and also cement mills. Smaller contribution of Ca^2+^ in frost can be explained by less “building” activity and the content of airborne particulate matter during the cool season (wet ground, frozen ground, snow cover). It is not also insignificant that calcium ions are bivalent. Additionally, the values of the nss Ca^2+^ / Ca^2+^ ratio in dew samples for all types of stations were close to 1, which results from nearly no limestone in Poland and confirmed the anthropogenic character of this pollution.

HCHO was found in 97 % of dew samples, with concentrations ranging from 0.010 to 5.40 meq/L. The highest average formaldehyde concentrations were observed for dew samples collected from the rural inland station (Wrocław). HCHO is emitted in to the atmosphere from exhaust gases from domestic heating, where it can be scavenged by wet and dry deposition. However, phenols were found in 95 % of dew samples, with concentrations ranging from 0.005 to 2.43 meq/L. The highest average sum of phenols concentrations was observed for dew samples collected from the urban coastal station. The high concentration levels of phenols in runoff waters collected in the area of the centre of Gdańsk and Gdynia are mainly caused by high traffic intensity and industrial factories situated in the surrounding area of these cities. The petroleum refinery alone introduces approximately 118 kg of phenols to the surface waters annually.

### 3.2. Correlation between concentrations

Seawater parameters, calculated as equivalent ratios, are respectively: Cl^-^/Na^+^ = 1.17; SO_4_^2-^/Na^+^= 0.12; K^+^/Na^+^= 0.022; Ca^2+^/Na^+^= 0.045; Mg^2+^/Na^2+^= 0.25. The average equivalent ratio of Cl^-^/Na^+^ in dew samples calculated in Table 4 was higher for all stations when compared with the appropriate seawater value. Very high values were observed for dew samples collected in Mława (max 57.6; mean 3.88) and Sopot (max 10.9; mean 4.92), where the concentration level of Cl^-^ was 3 to 4 times higher than Na^+^. A higher concentration of sodium ions (in comparison with chloride ions) was reported only in rural inland stations.

The average SO_4_^2-^/Na^+^ ratio in dew samples was always higher than the seawater ratio (urban coastal from 21 to 125 times; urban inland from 30 to 114; rural from 50 to 60) which can be attributed to emission from fuel combustion. The average K^+^/Na^+^ ratio in dew samples was also higher than the seawater ratio (for all stations from 30 to 61 times). The excess potassium can have different explanations. K^+^ is a major constituent of fertilizers and is generally present in windblown soil. Vegetation is another possible source for K^+^.

It was observed (Table 4) that the value of Ca^2+^/Na^+^ ratio in dew samples was generally much higher than that of seawater. The highest average value of Ca^2+^/Na^+^ ratio was noticed for urban inland agglomerations, such as: Mława (14.4) and Kraków (12.5). The Mg^2+^/Na^+^ ratio was almost equal to that of seawater in rural stations. The highest average values of the Mg^2+^/Na^+^ ratio were also observed for big urban agglomerations, such as Kraków (2.66), Sopot (1.97) and Gdańsk (1.54). For Mg^2+^, the primary source could be marine and the secondary could be continental.

**Figure 4.**
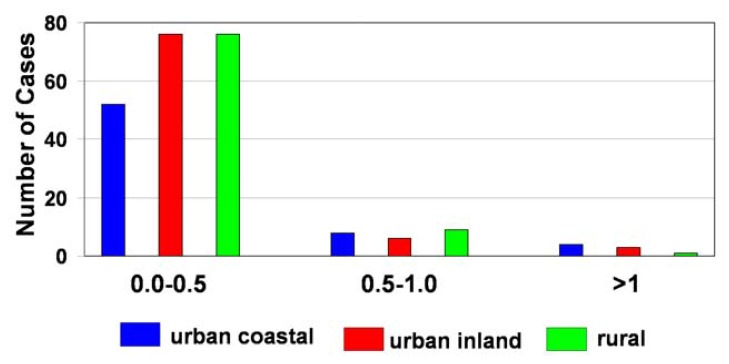
Frequency distribution of the equivalent ratio of NO_3_^-^/SO_4_^2^ in dew samples.

The frequency distributions of the NO_3_^-^/SO_4_^2-^ ratio for dew water for different forms of land-use in Poland are shown in Figure 4. The NO_3_^-^/SO_4_^2-^ ratio in dew samples ranged over a narrow interval, but in general the SO_4_^2-^ concentration was much higher than NO_3_^-^. A higher concentration of NO_3_^-^ in relation to SO_4_^2-^ was observed for 4 % of dew samples collected from urban areas.

**Figure 5.**
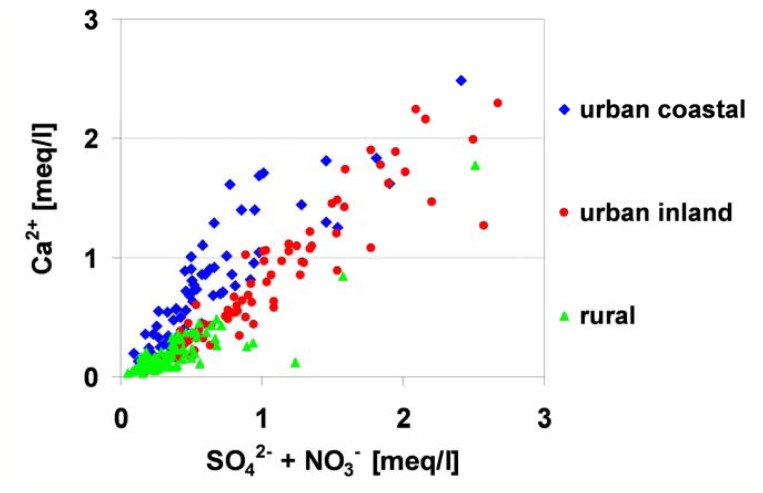
Relationships between Ca^2+^ and SO_4_^2-^ + NO_3_^-^ concentration in dew water.

The dew acidification process can be also explained by the relationship between the sum of acidifying anions, SO_4_^2-^ and NO_3_^-^, and alkaline cation Ca^2+^ (Figure 5). An increased concentration of Ca^2+^ ions in relation to the concentration of acidifying ions was observed especially in dew samples collected from the urban stations (inland and coastal). In rural areas, for obvious reasons, the concentration of the Ca^2+^ ion was lower. Together with an increasing concentration of the Ca^2+^ ion, the prevalence of acidifying ions was more noticeable.

**Figure 6.**
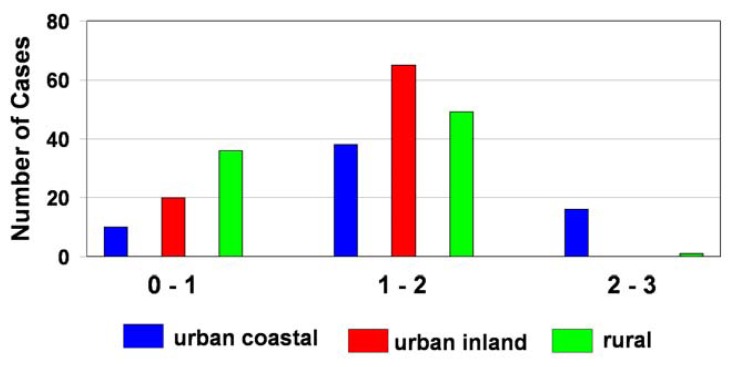
Frequency distribution of the equivalent ratio of Ca^2+^ + NH_4_^+^/ NO_3_^-^ + SO_4_^2-^ in dew samples.

The frequency distributions of the Ca^2+^ + NH_4_^+^/ NO_3_^-^ + SO_4_^2-^ ratio for different forms of land-use in Poland are shown in Figure 6. In the majority of cases, the values of this coefficient ranged from 0 to 2 (100 %, 99 % and 75 % of the obtained results for urban inland, rural inland and urban coastal stations, respectively). These values exceeded 2 only for urban coastal station (25 % of the obtained results), because of an excess of neutralizing ions (especially Ca^2+^).

### 3.3. The dew acidification process

The pH, which is a key measure of acid deposition in dew water, ranged from 5.22 to 7.35 in urban coastal stations, from 5.67 to 8.02 in urban inland stations and from 4.16 to 8.76 in rural inland stations. Acidic dew (pH<5.0) was observed in rural places twice (21.09.2006-4.16; 28.08.2006-4.30). However, strongly acidic dew (pH<4.0) was not seen. 57.4 % of the total number of dew samples were characterized by pH values above 6, and 34.5 % of them above 7.

**Figure 7.**
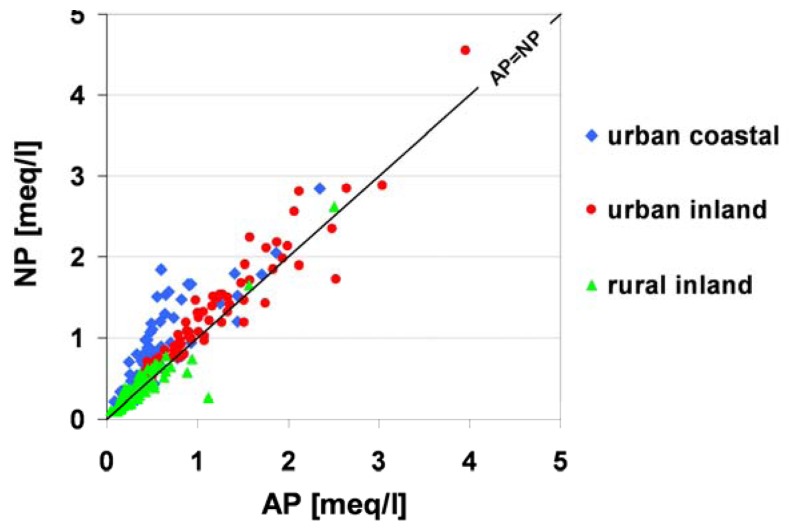
Relationship between AP and NP in dew water.

The acidification process of dew water can also be discussed using the relationship between AP (acidifying potential) and NP (neutralizing potential). Figure 7 illustrates the relationship between AP and NP in dew water for different types of land-use.

The theoretical curve, linking experimental data points, can be defined as a linear equation, whose general form is y=x (AP=NP). For the dew samples collected within the framework of this research project, linear equations: y=1.1x+0.26, r=0.841 (urban coastal stations); y=0.88x+0.22, r=0.937 (urban inland stations); y=0.92x+ 0.044 r=924 (inland stations) were calculated. It was estimated that a NP>AP relationship occurred for 88 % of the dew samples collected from coastal stations, whereas the opposite, AP>NP (acidic dew), was observed for 40 % of the samples collected from rural areas.

**Figure 8.**
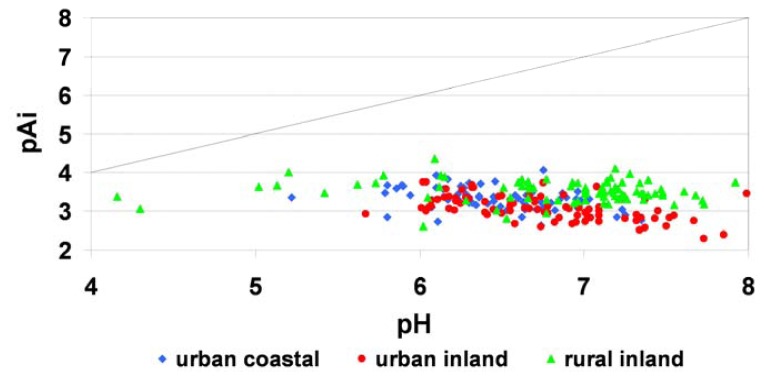
pAi against pH for dew samples from different types of landuse in Poland.

Hara *et al.* [26] suggest discussing dew chemistry from the viewpoint of an acid-base relationship and using the quantitative index pAi. The pAi is the hypothetical pH of atmospheric water if no neutralization takes place for both sulfic and nitric acids. These indexes focus only on acidic components, but pH is determined by the balance between an acidic and neutralizing component [30]. pAi values for dew water for different types of land-use in Poland ranged from 2.63 to 4.07 (urban coastal stations), from 2.30 to 3.76 (urban inland stations) and from 2.60 to 4.36 (rural inland stations; Figure 8).

**Figure 9.**
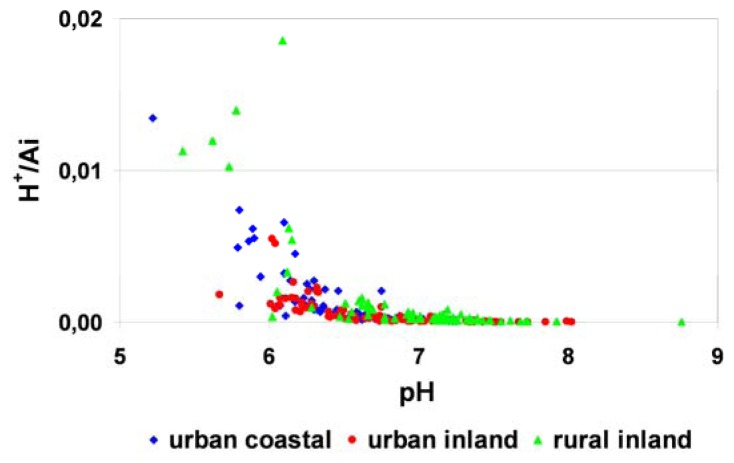
Fractional acidity, H^+^/Ai against pH for dew samples from different types of landuse in Poland.

For all of the samples, pAi values appeared in a highly limited range when compared with pH results. This suggests that pH was controlled by a basic species, which was further evidenced by plotting H^+^/Ai against pH in Figure 9, where pH values decreased with increasing fractional acidity, H^+^/Ai [26].

### 3.4. Ionic correlations between determined concentrations of analytes

In Table 5, correlation coefficients values obtained between determined analytes in collected dew samples are presented. Positive correlation coefficients are marked in bold. Correlation coefficients above 0.5 were observed for 64 %, 71 % and 49 % of the relationships for urban inland, urban coastal and rural inland stations, respectively. Values above 0.9 were observed for 16 %, 5.5 % and 7 % of the relationships for urban coastal, urban inland and rural stations, respectively. The strongest correlations were noticed between the sum of acidifying anions SO_4_^2-^ + NO_3_^-^ and Ca^2+^ ion for all urban and rural stations. Additionally, there was a strong correlation with Mg^2+^ for urban coastal stations (the influence of the vicinity of the Baltic Sea) and with NH_4_^+^ for rural stations (agricultural production). A very strong correlation was also observed for Na^+^ and Cl^-^ ions in urban coastal stations, as a natural consequence of the location of these stations close to the coast line. Regardless of the type of land-use, strong correlations between sulfate ion and cations, such as: Na^+^, NH_4_^+^, K^+^, Mg^2+^ were characteristic. In the case of urban coastal areas, such relationships were also observed for NO_3_^-^. In rural stations, relationships between NH_4_^+^ ion and anions, such as: Cl^-^, SO_4_^2-^, NO_3_^-^, PO_4_^3-^ played a more important role (such relationships were characteristic of agricultural areas, where phosphorous and ammonium fertilizers were used).

### 3.5. Dew chemistry in relation to meteorological data

#### 3.5.1. Type of air mass

Information regarding the chemical composition of dew is not often interpreted within the context of meteorological data. Obtained results concerning the chemical composition of dew water, collected in different places (rural and urban), were presented as a background for meteorological data-type for the air mass, character and direction of atmospheric circulation and horizontal pressure gradient.

Air mass is a large volume of air, in which prevailing meteorological conditions are generated by an exchange of energy and moisture between the atmosphere and its underlying surface over the area, from which the air mass flows. In about 60 % of the cases in Poland, polar maritime (Pm) masses flow from the west. They are formed over the North Atlantic and are often accompanied by atmospheric precipitation. Less frequently observed are the polar-continental (Pc) masses flowing from Eastern Europe, which are dry as well as cold during winter and dry as well as hot during summer. Arctic air masses (PA) flow occasionally from the Arctic Sea and bring cold weather with possible showers. That is reason limited number of the days with arctic air mass and even lack of dew chemical composition in case of arctic air mass for rural in land.

**Figure 10.**
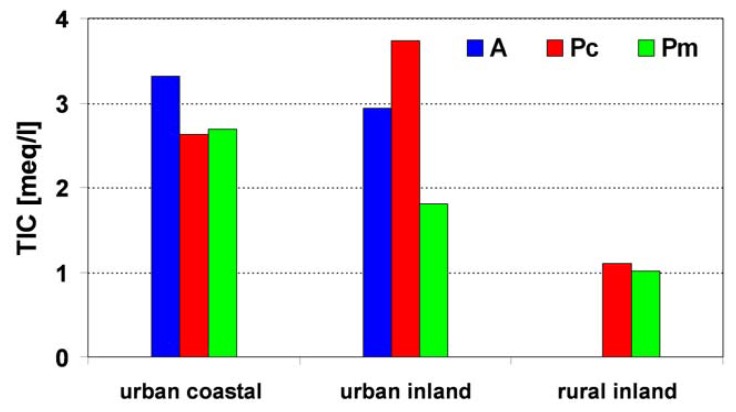
TIC in dew samples versus type of air mass (A-arctic; Pc-polar continental; Pm-polar maritime).

For each day with dew deposition, the type of the flowing air mass over Poland was defined on the basis of sea-level synoptic maps. The diversity of chemical composition of dew, in relation to the air mass type, is presented in Table 6 and Figure 10.

In rural stations, dew pollution, represented by obtained TIC results, did not differ significantly between flowing air masses, reaching values of 1.11 and 1.01 meq/L for Pc and Pm masses, respectively. It should be explained in such a way that there are not so many local pollution sources influencing the near-ground air layers in rural areas in comparison with urban ones. In this type of station, in a TIC structure, an increased contribution of NH_4_^+^ from agricultural activity was observed.

The inner diversity of dew pollution in the urban stations was closely related to its distance from the Baltic coast. It was a characteristic feature that average TIC results in A, Pc and Pm masses in urban coastal stations were comparable, reaching the values of 3.32, 2.63 and 2.69 meq/L, respectively. However, these TIC results in urban inland stations showed significant diversity, reaching the values of 2.94, 3.73 and 1.81 meq/L, respectively. This can be explained by local breeze circulation in the warm part of the year, mainly in the form of a diurnal breeze. Breeze circulation, generated by the thermal difference between land and sea with a prevailing low horizontal pressure gradient, occurred in each of the investigated air mass types. A diurnal breeze forces air flown in from the sea onto land, causing on increase in the concentration of Na^+^ and Cl^-^ ions. The flowing air mass is then polluted by local emission sources.

Taking into consideration both the smaller thermal contrast between land and sea during the night than during the day and the shortness of the night in the warm part of the year, the night breeze was not marked (as well) as the diurnal one. This means that a considerably smaller volume of air (within the atmospheric boundary layer) was transferred from land to sea during the night than in the opposite direction during the day. Dew in urban coastal regions had constituents, which were both of maritime origin as well as emitted from local sources, and the pollutant concentration was relatively high.

The highest TIC values were observed in urban coastal stations in the case of arctic air mass, which flows generally from the north sector, from the direction of local emission sources situated in the whole Tricity agglomeration. The mechanism of local breeze circulation, which was described above, did not occur in inland areas and therefore TIC diversity between air masses in urban inland stations was higher.

In rural and urban inland stations, the highest pollutant concentrations were observed for dew samples collected during the day, when meteorological conditions were influenced by Pc masses. In rural areas, the difference between Pc and Pm masses reached only 10 %. In the stations representing urban conditions, TIC values in Pc mass were more than twice as high as the Pm value. An explanation seems to be quite obvious. In comparison with Pm and A masses, dry air flow of Pc mass was characterized by lack of precipitation or small rainfalls with a limited range. This considerably limited the removal rate of pollutants (aerosols, dust or gaseous components) from air and caused an increase in the significance of their transport. In inland areas, a considerably higher efficiency of pollutants' removal from the atmosphere occurred in Pm and A masses, in which precipitation was more abundant, when compared with Pc mass. Moreover, during the nights which followed the days with the Pc mass, the near-ground inversion layer was characteristic, above which the second inversion layer often occurred due to the mesoscale subsidence of air in anticyclone systems. Under such conditions, there was a tendency for pollutants to accumulate in the sub-inversion layer (an average thickness of a few hundred meters above the ground) due to the considerably limited possibilities of air mixing. Noticeably smaller differences in TIC values between Pc and Pm masses in rural stations, as compared with measuring sites situated in urban inland terrains, additionally confirmed a low level of atmospheric pollution in rural areas.

#### 3.5.2. Synoptic situation

Quite important, in the context of the level of dew pollution, is the height of the mixing layer and the type of thermal stratification occurring within it. The efficient vertical mixing of air occurs under unstable conditions. Pollutants are more efficiently transported within the mixing layer than in other types of thermal stratification. Additionally, the depth of the mixing layer is the greatest. Under both: stable and temperature inversion conditions, the vertical transport of pollutants between air layers is significantly limited. Neutral stratification and the potential instability with the prevalence of ascending air currents are characteristic for low pressure systems during the whole year. In anticyclones, stable stratification connected to an inversion of radiation or subsidence origin is dominant in the atmospheric boundary layer, particularly in the cold part of the year. The occurrence of stable stratification considerably limits vertical air exchange allowing for a high and steady concentration level of emitted atmospheric pollutants. For each day with dew, on the basis of sea level synoptic maps (00 GMT) [31], one of three typical synoptic situations: anticyclonic (AS), cyclonic (CS) or transition (TS), determining the intermediate situation between AS and CS, was assigned.

**Figure 11.**
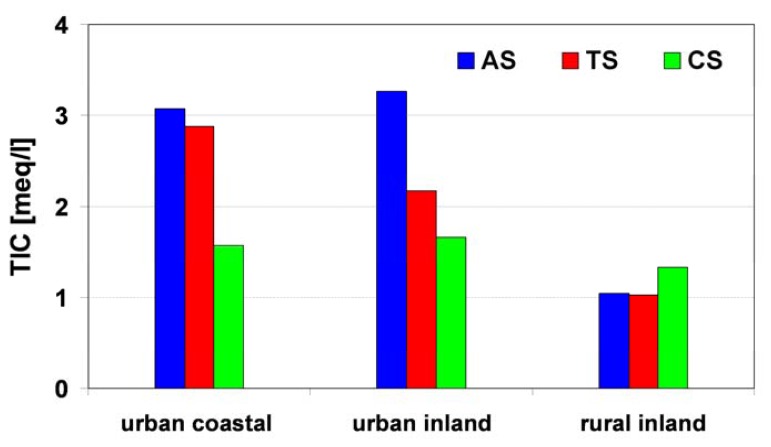
TIC in dew samples versus synoptic situation (AS-anticyclonic; CS-cyclonic; TS-transitional).

Conditions allowing for dew formation occured in the anticyclonic situation, when Poland was surrounded by the central part of a high pressure system. Then, the prevailing macroscale processes of air subsidence conduced to cloudless weather with a very weak wind. A lesser probability of dew generation refers to the transition situation and in a greater degree to the cyclonic one. It finds its reflection in the population of the investigated cases of dew formation (AS-168, TS-37, CS-30, respectively). The diversity of the chemical composition of the dew in relation to the synoptic situation is presented in Table 7 and Figure 11.

In rural stations, average TIC results were the lowest and characterized by the smallest diversity for AS, TS and CS situations, reaching values of 1.04, 1.02 and 1.33 meq/L, respectively (Figure 11). In relation to AS and TS situations, these values were two times lower when compared with stations representing urban areas. The smallest TIC diversity for three investigated landuse categories was characteristic for cyclonic situations. It confirmed the significantly smaller role of local pollutant emission sources under such conditions. The reason lies in the specificity of the cyclonic weather. Then, the mesoscale ascending flow occurred, which caused an intensive vertical air exchange and dispersion of pollutants. Consequently, the role of local emission sources decreased and pollutant concentrations in dew in all types of stations was comparable to the (macro) regional background. Additionally, the washout of pollutants from the atmosphere in cyclonic weather was more efficient than in the anticyclonic one, due to more frequent precipitation events.

In some way it was surprising that in stations representing rural areas in AS situations, pollutant concentrations in dew was around 30 % lower than within CS days. Taking into account the cyclonic weather and small emission from local sources, long-range transport with a regional background, which was the main cause of increased Cl^-^ and NH_4_^+^concentration, played a more important role in TIC formation.

For both coastal and inland urban stations, distribution of TIC values was opposite of those in rural areas. These results were highest during days with AS weather, a little smaller for days with TS situations and the smallest for cyclonic conditions (CS). In anticyclonic weather, there was a lack of precipitation and thermal inversion, which had a limited thickness and often occurred during the night (when dew is generated). Under such conditions, pollutants from near-ground emission sources accumulated. As a result, during days with a high pressure system, pollutant concentrations in dew increased, and the highest TIC diversities between rural and urban areas were observed.

**Figure 12.**
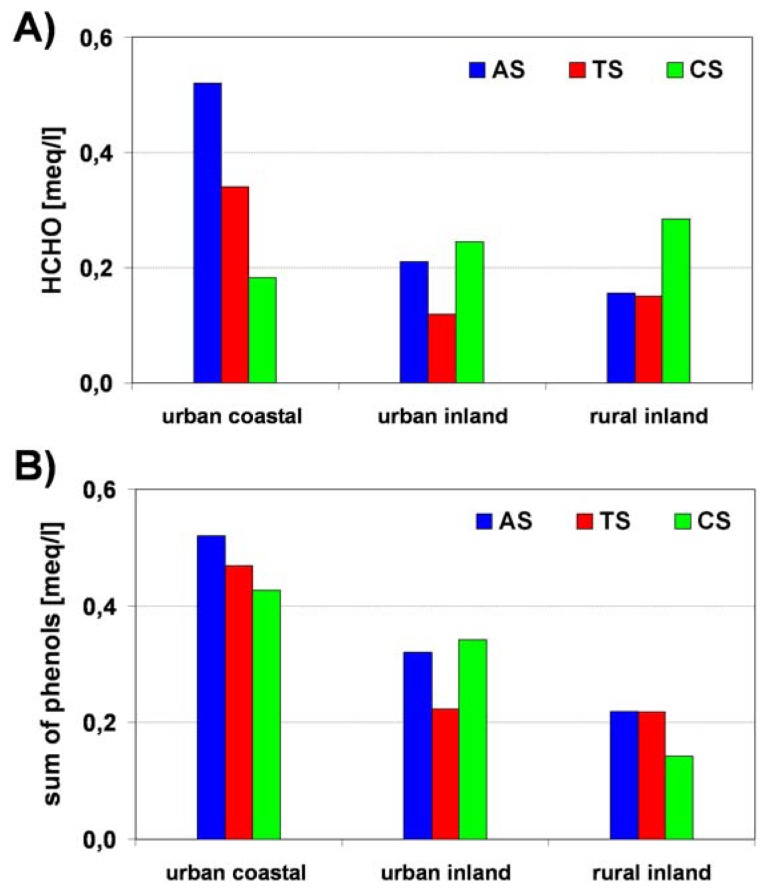
Concentration of HCHO (A) and sum of phenols (B) in dew samples versus synoptic scale atmospheric circulation.

The intercorrelation of formaldehyde with pollutant components (NH_4_^+^, nss SO_4_^2-^, NO_3_^-^) suggested a possible anthropogenic input for HCHO, which was found in 97 % of analyzed samples. The most likely pollutant source is incomplete oxidation of hydrocarbons released by fossil fuel combustion, including industrial and domestic heating and motor vehicle traffic. There was a lack of significant correlations among the concentration levels of HCHO and phenols in relation to atmospheric circulation (Figure 12). The highest concentrations were noticed in dew samples collected from the Tricity agglomeration within days when anticyclonic circulation occurred. The obtained data confirmed the same relations for TIC analysis versus atmospheric circulation, which were described earlier.

#### 3.5.3. Direction of atmospheric circulation

An important factor influencing air pollutant concentrations is the direction of airflow. The near ground wind direction is highly changeable because of existing turbulence and local effects imposed on synoptic scale airflow. In this paper, the direction of airflow was determined on the basis of sea level synoptic maps and generalized to eight directions in which the wind rose for each night with dew collection and for the whole territory of Poland.

**Figure 13.**
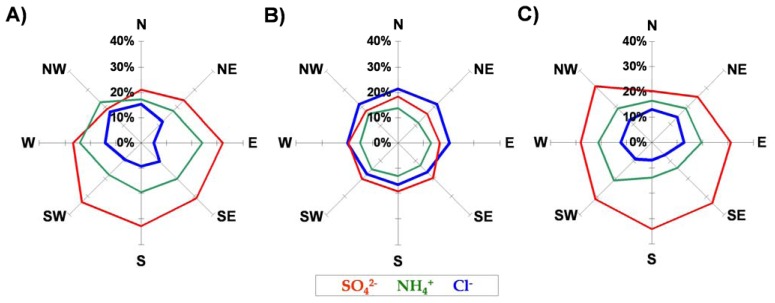
Relative contribution of SO_4_^2-^, NH_4_^+^ and Cl^-^ in dew samples versus direction of circulation for different types of landuse in Poland (A-rural station Dziemiany; B-urban coastal stations Tricity; C-urban inland station Bytów).

There was no clear relation among select ion concentrations and different directions of atmospheric circulation, which could possibly be attributed to the emission background of the selected sampling sites (Figure 13). Nevertheless, some effects were visible. A directional asymmetry of major ions was observed at urban coastal stations where Cl^-^ had the highest concentration when air flew from the sea (northern half of horizon) while SO_4_^2-^ took this role during southern circulation, when air masses came from inland areas. On the other hand, all Cl^-^ concentrations at urban coastal stations, independent of circulation direction, were around twice as large as those at urban inland stations. This fact supports the hypothesis about the significant role of a diurnal sea breeze, which penetrates deep inland, delivering sea-salt aerosol, even on days with synoptic scale atmospheric circulation from a continental interior.

Stations located inland (both urban and rural) presented a higher concentration of Cl^-^ during airflow from NW-N-NE directions with a minimum observed on circulation from the SE half of the horizon. The opposite pattern was visible in the case of sulphates, which came probably from distant industrialized areas further in the south. No discernible trend was detected for NH_4_^+^, which was emitted mainly from dispersed agricultural sources, with no clear directional preferences.

**Figure 14.**
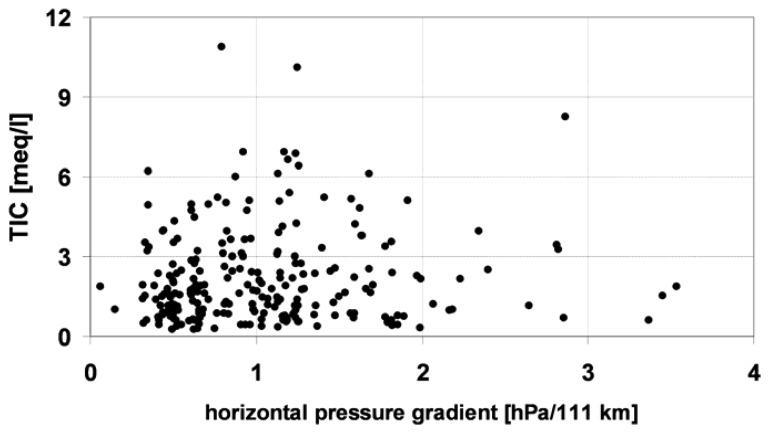
Concentration of TIC in dew samples versus horizontal pressure gradient.

Figure 14 illustrated the relation between TIC values and horizontal pressure gradient, the latter being an index of atmospheric circulation intensity. The total amount of TIC showed a weak dependence on the horizontal pressure gradient. The maximum TIC values were observed for the moderate pressure gradient (0.7-1.7 hPa/111 km), whereas above and below this parameter, TIC values were lower.

This can be caused by an insufficient air pollution supply to the near-ground layer of air during nocturnal atmospheric calms caused by a low horizontal pressure gradient (below 0.7 hPa/111 km). On the other hand, when the horizontal pressure gradient was high (more than 1.7 hPa/111 km) pollution was dispersed by turbulence in vigorous airflow, which resulted in a lowering of observed concentrations. If the pressure gradient was moderate (0.7-1.7 hPa/111 km), the observed airflow brought pollution from neighbouring areas but was not strong enough to cause intense dispersion of pollutants, resulting in lowered concentrations. Nevertheless, the relationships presented are not unequivocal because of the role of other factors controlling dew formation and chemistry.

## 4. Conclusions

Dew water in Poland was characterised by a relatively high concentration of major ions with an average TIC 2.46 meq/L, when compared with precipitation (wet only; 0.37 meq/L) or orographic fog (1.01 meq/L) albeit very similar to hoarfrost (2.86 meq/L). Due to a high concentration of chemical constituents, dew is particularly important in pollutant deposition processes. Hence, over the large area of European lowlands, dew (together with hoarfrost) form an important path of pollutants' flux to the ground, being responsible at least for an additional 50% deposition through atmospheric precipitation. Ion concentrations in precipitation is 6-7 times lower in comparison with dew, but the amount of precipitation is a dozen times higher.

A specific feature of dew chemistry was a low level of NO_3_^-^ when compared with precipitation, presumably caused by a decrease of traffic emission when dew was formed. The dominant ions were SO_4_^2-^ at rural and inland urban stations and Ca^2+^ at urban coastal stations. The discernible decrease of the marine components was clearly visible with an increasing distance away from the Baltic coast. Because of high concentration of both Ca^2+^ and NH_4_^+^, pH values of dew water were usually high.

The chemistry of dew water was strongly influenced by atmospheric circulation and air mass character. The highest pollutant concentrations in dew occurred when there was a lack of precipitation in the advecting air mass and weak synoptic- or local- scale airflow was observed supplying the area of dew formation with pollutants emitted from local sources. Local breeze circulation appeared to be very important for both pollutant concentration and composition in coastal areas.

Taking into account both high TIC values and the annual water equivalent, dew was a considerable factor of wet deposition, being responsible for an additional 60% of pollutants input into the ground when compared with precipitation.

## Figures and Tables

**Figure 2.**
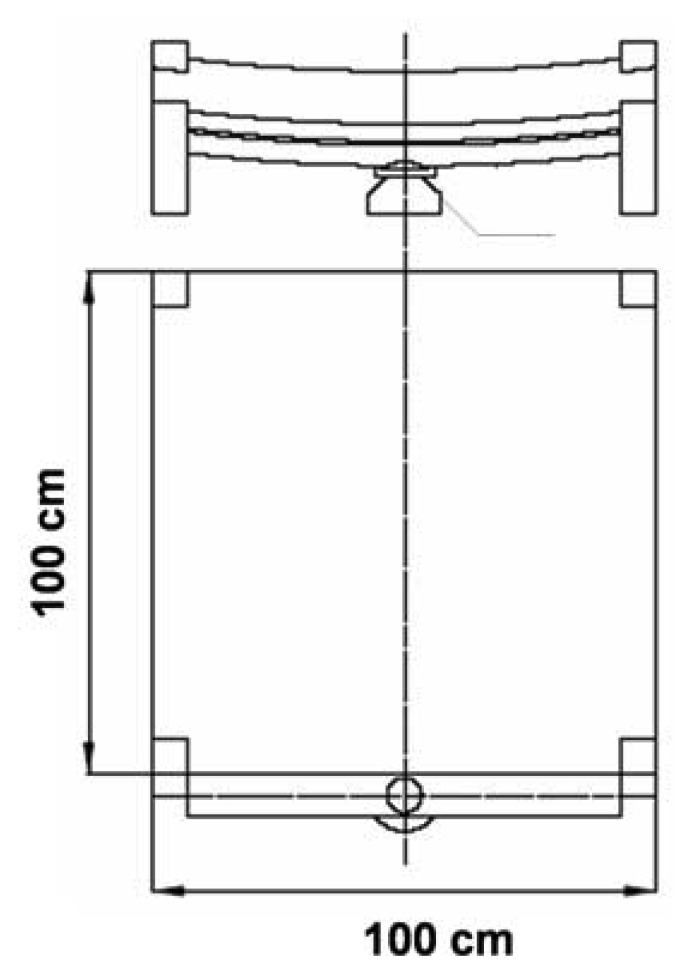
Sampler used for the collection of dew samples.

**Table 1.** Detailed characteristics of the sampling sites.

**Sampling site location**	**Geographic coordinates**	**Elevation [m a.s.l.]**	**Type of area**	**Landform type**	**Site description**
**Coastal urban sites**					
Gdańsk	54° 20″ N 18° 36″ E	74	suburban	gentle slope	200 m away from the main road connecting the Gdańsk centre with the Tricity ring road; 3 km WSW of the Gdańsk city centre and 8 km away from the Gdańsk Gulf; 460,000 citizens
Sopot	54° 27″ N 18° 34″ E	5	urban	flat	intersection close to the city centre; in the vicinity of the coast line (200 m), surrounded directly by allotments and a residential area; 40,500 citizens
Gdynia	54° 29″ N 18° 32″ E	37	urban	flat	intersection close to the city centre with very intensive road transport; close to the coast line (1.5 km), surrounded by a residential area; 253,500 citizens
**Inland urban sites**					
Mława	53° 08″ N 20° 21″ E	142	urban	flat	outskirts of the country town; sampling point located in the vicinity of the allotments and a building with flats; 30,000 citizens
Kraków	50° 07″ N 19° 55″ E	225	suburban	flat	sampling point surrounded by allotments and a building with flats; 757,500 citizens
Bytów	54° 10″ N 17° 30″ E	147	urban	flat	outskirts of the city centre; only single-family buildings; 23,500 citizens
**Rural sites**					
Dziemiany	54° 00″ N 17° 46″ E	166	rural, lake district and foresty	hilly	village located on the outskirts of the Wdzyński Landscape Park; surrounded by a forest; 1,600 citizens
Wrocław	51° 07″ N 17° 05″ E	116	rural	Flat (mikro concave)	Meteorological Observatory; measuring site situated on the east of Wrocław, 5 km from the centre; located close to the Odra river and Szczytnicki park, surrounded directly by allotments

**Table 2.** Information concerning the sampler applied to the collection of dew samples.

**Characteristics of the sampler**	**Size of the sampler**
the collecting surface of this sampler was made of rigid polyethylene foil mounted on a wooden frame	100 cm × 100 cm (Figure 2)
the sampler was mounted at a 30-degree angle to promote the flow of condensation droplets to a collector (groove) and subsequently to a collection vessel (50 cm^3^ flask)	
to increase the efficiency of collection, the sampler has been thermally isolated from the ground with 15-cm-thick polystyrene foam	

**Table 3.** Summary characteristics of analytical techniques used in the study.

**Analyte**	**Technique**	**Analytical parameters**	**Limit of detection [mg/L]**	**Precision [% RSD]**
Anions	IC	AS9-HC column (2 × 250 mm),	Br^-^, F^-^, Cl^-^,	1
		AutoSuppression Recycle Mode	NO_3_^-^, SO_4_^2-^= 0.01	
		ASRC^®^-ULTRA (2 mm), conductivity	NO_2_^-^=0.05	
		detection, eluent 9.0 mM Na_2_CO_3_,	PO_4_^3-^= 0.04	
		flow rate 0.25 ml/min		
Cations		CS12A column (2 × 250 mm),	0.01	
		AutoSuppression Recycle Mode CSRS		
		^®^-ULTRA (2 mm), conductivity		
		detection, eluent 20 mM		
		Methanesulfonic Acid,		
		flow rate 0.25 ml/min		
Phenols	photometry	Absorbance measured at 495 nm	0.001	5
HCHO		Absorbance measured at 585 nm	0.005	5

**Table 4.** Miniumum, maximum and average analyte concentrations (meq/L and μS/cm-conductivity) and selected hydrochemical indices in dew samples.

**ANALYTES**	**STATS**	**URBAN COASTAL**			**URBAN INLAND**			**RURAL INLAND**	

		**Gdańsk**	**Gdynia**	**Sopot**	**Bytów**	**Kraków**	**Mława**	**Dziemiany**	**Wrocław**

**1**	**2**	**3**	**4**	**5**	**6**	**7**	**8**	**10**	**11**
N		13	31	20	36	19	29	33	53

**Conductivity**	max	288	482	747	518	881	298	276	396
	mean	151	151	183	171	337	152	92. 0	56.7
	min	28.8	31.2	35.1	29.2	56.2	69.5	19.2	20.2
	f (%)	100	100	100	100	100	100	100	100

**pH**	max	7.20	7.35	7.03	7.99	8.02	7.32	8.76	7.47
	mean	6.31	6.52	6.55	6.80	7.12	6.49	7.15	6.63
	min	5.22	5.80	5.94	6.01	6.57	5.67	5.62	4.16
	f (%)	100	100	100	100	100	100	100	100

**Na^+^**	max	0.88	1.07	0.26	0.68	0.22	0.34	0.96	0.22
	mean	0.34	0.34	0.13	0.15	0.11	0.14	0.11	0.043
	min	0.013	0.024	0.034	0.020	0.040	0.0030	0.026	0.0052
	f (%)	100	100	100	100	100	100	100	100

**NH_4_^+^**	max	0.45	1.18	0.42	0.60	0.59	0.70	0.84	0.82
	mean	0.25	0.23	0.27	0.33	0.29	0.35	0.26	0.15
	min	0.063	0.029	0.085	0.15	0.067	0.10	0.084	0.039
	f (%)	100	35.5	100	100	100	100	100	100

**K^+^**	max	0.37	0.43	0.17	0.26	0.36	0.22	0.17	0.16
	mean	0.15	0.13	0.092	0.077	0.12	0.086	0.052	0.046
	min	0.029	0.0095	0.037	0.025	0.064	0.019	0.018	0.0067
	f (%)	100	100	100	100	100	100	100	100

**Mg^2+^**	max	0.45	0.77	0.32	0.62	1.14	0.46	0.15	0.18
	mean	0.23	0.21	0.072	0.074	0.29	0.093	0.028	0.031
	min	0.026	0.032	0.012	0.0025	0.012	0.011	0.0025	0.00083
	f (%)	100	100	100	100	100	100	100	100
**Ca^2+^**	max	1.81	2.49	1.40	2.80	4.43	2.16	1.78	0.84
	mean	0.89	0.74	0.75	0.73	1.33	0.68	0.27	0.16
	min	0.13	0.086	0.15	0.037	0.15	0.16	0.027	0.030
	f (%)	100	100	100	100	100	100	100	100
**F^-^**	max	0.10	0.056	0.020	0.042	0.039	0.062	0.22	0.25
	mean	0.018	0.012	0.010	0.016	0.021	0.011	0.036	0.015
	min	0.0016	0.0016	0.0016	0.00053	0.0042	0.0016	0.0042	0.00053
	f (%)	76.9	71.0	100	94.6	100	100	100	83.0
**Cl^-^**	max	1.14	1.71	1.00	0.97	0.84	0.77	0.23	0.34
	mean	0.45	0.55	0.54	0.20	0.23	0.26	0.13	0.067
	min	0.065	0.062	0.27	0.027	0.052	0.059	0.065	0.0037
	f (%)	100	100	100	100	100	100	100	100
**NO_2_^-^**	max	0.070	0.21	0.052	0.31	0.16	0.049	0.13	0.048
	mean	0.030	0.035	0.028	0.054	0.052	0.023	0.012	0.014
	min	0.0041	0.0035	0.0048	0.0024	0.0072	0.0071	0.0015	0.0028
	f (%)	76.9	67.7	55.0	86.5	47.4	51.7	81.8	100
**NO_3_^-^**	max	0.72	0.47	0.54	0.81	0.34	0.51	1.74	0.50
	mean	0.16	0.12	0.12	0.15	0.12	0.18	0.12	0.062
	min	0.0066	0.026	0.032	0.00097	0.0082	0.012	0.013	0.015
	f (%)	100	100	100	100	94.7	96.6	100	100
**PO_4_^3-^**	max	0.40	0.47	0.090	0.057	0.12	0.13	0.24	0.12
	mean	0.092	0.10	0.031	0.020	0.049	0.044	0.034	0.019
	min	0.0091	0.0044	0.0073	0.0075	0.0059	0.012	0.0047	0.0028
	f (%)	61.5	74.2	75.0	35.1	63.2	69.0	51.5	26.4
**SO_4_^2-^**	max	1.40	1.94	0.87	4.46	3.77	1.71	1.22	1.07
	mean	0.72	0.48	0.40	0.95	1.37	0.68	0.40	0.25
	min	0.067	0.030	0.15	0.11	0.34	0.30	0.030	0.065
	f (%)	100	100	100	100	100	100	100	100
**SO_4_^2-^ + NO_3_^-^**	max	1.91	2.41	0.95	5.10	3.98	2.16	2.51	1.58
	mean	0.88	0.60	0.52	1.10	1.49	0.85	0.52	0.31
	min	0.12	0.091	0.19	0.18	0.38	0.42	0.048	0.082
**Cl^-^ / Na^+^**	max	8.42	6.55	10.9	4.18	3.91	57.6	4.63	7.02
	mean	2.29	2.48	4.92	1.64	1.98	3.88	2.15	1.86
	min	0.32	0.80	1.68	0.21	0.83	0.67	0.086	0.059
**SO_4_^2-^ / Na^+^**	max	17.3	8.61	8.55	14.6	29.0	287	14.5	24.5
	mean	3.94	2.56	3.64	6.72	13.7	15.0	5.99	7.20
	min	1.04	0.25	0.93	1.56	5.52	1.48	0.91	2.18
**K^+^ / Na^+^**	max	4.12	3.81	1.16	1.45	3.44	6.23	2.86	2.50
	mean	1.01	0.70	0.77	0.68	1.35	0.83	0.75	1.21
	min	0.065	0.13	0.52	0.14	0.49	0.26	0.093	0.30
**Ca^2+^ / Na^+^**	max	16.5	14.4	18.8	12.3	24.1	279	17.6	30.5
	mean	4.57	4.45	6.65	4.81	12.5	14.4	4.38	5.41
	min	1.01	0.55	1.36	0.75	2.42	2.12	0.12	0.87
**Mg^2+^ / Na^+^**	max	8.99	2.37	2.36	1.52	6.53	40.5	1.27	2.08
	mean	1.54	0.88	0.63	0.40	2.66	1.97	0.33	0.70
	min	0.090	0.20	0.12	0.086	0.19	0.14	0.048	0.033
**NO_3_^-^ / SO_4_^2-^**	max	0.83	2.08	2.95	0.86	0.30	1.67	2.25	0.88
	mean	0.27	0.39	0.39	0.24	0.099	0.33	0.26	0.27
	min	0.029	0.086	0.069	0.0024	0.013	0.022	0.013	0.068
**Ca^2+^ + NH_4_^+^ / NO_3_^-^ + SO_4_^2-^**	max	1.72	2.84	2.77	1.44	1.31	1.54	2.34	1.72
	mean	1.03	1.45	2.00	1.09	1.08	1.21	1.17	1.03
	min	0.69	0.84	1.31	0.64	0.93	0.94	0.24	0.65
**Σ anions / Σ cations**	max	0.99	0.99	1.39	1.21	0.94	1.06	1.28	1.31
	mean	0.86	0.85	0.87	0.97	0.85	0.87	0.97	0.99
	min	0.80	0.80	0.80	0.88	0.80	0.80	0.86	0.85
**TIC**	max	6.13	8.24	3.77	10.1	10.9	6.87	6.20	3.79
	mean	3.08	2.77	2.43	2.73	3.93	2.51	1.43	0.83
	min	0.39	0.62	0.88	0.52	0.97	1.18	0.32	0.27
**PDI [% ]**	max	11.1	11.1	16.3	9.51	10.8	11.1	12.4	13.4
	mean	7.48	8.13	9.11	4.12	8.18	7.15	4.43	4.25
	min	0.65	0.49	0.84	0.019	2.84	0.19	0.22	0.34
**pAi**	max	3.92	4.07	3.74	3.76	3.42	3.38	4.36	4.10
	mean	3.19	3.37	3.33	3.15	2.88	3.11	3.39	3.60
	min	2.73	2.63	3.03	2.30	2.40	2.67	2.60	2.81
**AP**	max	1.86	2.34	0.93	5.05	3.95	2.12	2.50	1.56
	mean	0.84	0.56	0.50	1.08	1.47	0.83	0.50	0.30
	min	0.12	0.086	0.18	0.17	0.38	0.42	0.044	0.079
**NP**	max	2.05	2.85	1.67	3.26	4.54	2.81	2.62	1.65
	mean	0.93	0.81	1.02	1.06	1.61	1.03	0.53	0.31
	min	0.13	0.19	0.28	0.22	0.37	0.46	0.11	0.090
**NP / AP**	max	1.83	3.09	2.88	1.46	1.32	1.59	2.51	1.73
	mean	1.07	1.55	2.05	1.11	1.08	1.23	1.19	1.05
	min	0.71	0.86	1.34	0.65	0.93	0.95	0.23	0.65
**loss Mg^2+^**	max			0.017	0.019		0.0069	0.17	0.011
	mean	0.062	0.014	0.011	0.0045	0.0026	0.0037	0.023	0.0049
	min			0.0023	0.00027		0.0015	0.00037	0.0018
**nss SO_4_^2-^**	max	1.38	1.87	0.85	4.41	3.74	1.67	1.11	1.06
	mean	0.67	0.44	0.39	0.93	1.36	0.66	0.38	0.24
	min	0.065	0.024	0.14	0.11	0.34	0.28	0.026	0.062
**nss Ca^2+^**	max	1.80	2.46	1.39	2.78	4.42	2.14	1.77	0.84
	mean	0.87	0.73	0.75	0.73	1.32	0.67	0.27	0.16
	min	0.13	0.082	0.15	0.036	0.15	0.15	0.026	0.029
**loss Mg^2+^/ Mg^2+^**	max			0.89	1.67		0.69	3.81	5.96
	mean	1.55	0.14	0.57	0.49	0.23	0.29	1.07	1.77
	min			0.052	0.040		0.080	0.034	0.23
**nss SO_4_^2-^ SO_4_^2-^**	max	0.99	0.99	0.99	0.99	1.00	1.00	0.99	1.00
	mean	0.94	0.90	0.95	0.97	0.99	0.97	0.97	0.98
	min	0.88	0.52	0.87	0.92	0.98	0.92	0.87	0.94
**nss Ca^2+^ / Ca^2+^**	max	1.00	1.00	1.00	1.00	1.00	1.00	1.00	1.00
	mean	0.98	0.98	0.99	0.98	0.99	0.99	0.97	0.99
	min	0.96	0.92	0.97	0.94	0.98	0.98	0.64	0.95
**HCHO**	max	0.98	3.15	0.25	1.71	0.27	0.410	2.12	5.40
	mean	0.40	0.76	0.13	0.29	0.13	0.15	0.260	1.22
	min	0.050	0.060	0.050	0.010	0.050	0.060	0.040	0.100
	f (%)	90.9(11)*	82.8 (29)*	100 (19)*	100 (34)*	100 (18)*	100 (27)*	100 (32)*	100 (51)*
**Sum of phenols**	max	0.83	2.43	0.57	1.51	0.22	0.55	0.470	1.76
	Mean	0.58	0.89	0.22	0.55	0.11	0.16	0.084	0.299
	min	0.37	0.10	0.060	0.020	0.058	0.035	0.033	005
	f (%)	100 (7)*	73.9 (23)*	100 (15)*	96.8 (31)*	94.4 (18)*	100 (23)*	100. (32)*	96.1(51)*

f – frequency of occurence

**Table 5.** Ion pair correlations.

	**F^-^**	**Cl^-^**	**NO_3_^-^**	**PO_4_^3-^**	**SO_4_^2-^**	**Na^+^**	**NH_4_^+^**	**K^+^**	**Mg_2_^+^**	**Ca^2+^**	**SO_4_^2-^ +NO_3_^-^**

**URBAN COASTAL**												
**F^-^**	1.00										
**Cl^-^**	0.48	1.00									
**NO_3_^-^**	**0.61**	**0.53**	1.00								
**PO_4_^3-^**	-0.20	**0.64**	-0.21	1.00							
**SO_4_^2-^**	**0.75**	**0.76**	**0.84**	0.083	1.00						
**Na^+^**	0.44	**0.92**	**0.58**	**0.61**	**0.79**	1.00					
**NH_4_^+^**	-0.042	**0.70**	-0.076	**0.90**	0.21	**0.63**	1.00				
**K^+^**	**0.55**	**0.88**	**0.72**	0.41	**0.88**	**0.97**	0.48	1.00			
**Mg^2+^**	**0.67**	**0.60**	**0.83**	0.00039	**0.94**	**0.73**	0.11	**0.84**	1.00		
**Ca^2+^**	**0.74**	**0.71**	**0.87**	-0.045	**0.93**	**0.65**	0.042	**0.76**	**0.80**	1.00	
**SO_4_^2-^ + NO_3_^-^**	**0.74**	**0.73**	**0.90**	0.023	**0.99**	**0.76**	0.15	**0.87**	**0.94**	**0.94**	1.00

**URBAN INLAND**												

**F^-^**	1.00										
**Cl^-^**	0.33	1.00									
**NO_3_^-^**	0.44	0.30	1.00								
**PO_4_^3-^**	-0.14	0.34	-0.074	1.00							
**SO_4_^2-^**	**0.64**	**0.57**	0.33	0.23	1.00						
**Na^+^**	0.45	**0.82**	0.41	0.074	**0.60**	1.00					
**NH_4_^+^**	**0.55**	0.31	0.36	-0.026	**0.54**	0.34	1.00				
**K^+^**	**0.54**	**0.58**	0.43	0.21	**0.80**	**0.76**	0.48	1.00			
**Mg^2+^**	0.32	**0.52**	0.13	**0.59**	**0.73**	0.42	0.13	**0.62**	1.00		
**Ca^2+^**	**0.60**	**0.65**	0.46	0.30	**0.96**	**0.62**	0.44	**0.78**	**0.73**	1.00	
**SO_4_^2-^ + NO_3_^-^**	**0.68**	**0.58**	**0.54**	0.18	**0.97**	**0.63**	**0.57**	**0.82**	**0.68**	**0.97**	1.00

**RURAL**												

**F^-^**	1.00										
**Cl^-^**	0.36	1.00									
**NO_3_^-^**	0.050	0.16									
**NO_3_^-^**	**0.83**	0.32	1.00								
**PO_4_^3-^**	**0.68**	0.18	**0.75**	1.00							
**SO_4_^2-^**	0.32	0.49	**0.59**	0.25	1.00						
**H^+^**	-0.17	-0.30	0.055	-0.11	0.40						
**Na^+^**	0.044	0.49	0.044	-0.023	**0.53**	1.00					
**NH_4_^+^**	**0.70**	**0.55**	**0.86**	**0.58**	**0.75**	0.20	1.00				
**K^+^**	0.46	0.49	**0.67**	0.47	**0.76**	0.45	**0.83**	1.00			
**Mg^2+^**	0.18	0.50	0.32	0.31	**0.54**	0.30	0.31	0.45	1.00		
**Ca^2+^**	**0.80**	0.44	**0.97**	**0.73**	**0.63**	0.082	**0.83**	**0.60**	0.42	1.00	
**SO_4_^2-^ + NO_3_^-^**	**0.73**	0.42	**0.95**	**0.65**	**0.81**	0.23	**0.91**	**0.78**	0.44	**0.94**	1.00

**Table 6.** Dew chemistry in relation to the type of air mass (A-arctic; Pc-polar continental; Pm-polar maritime).

**Land use**	**Type of air mass**		**pH**	**Conductivity [μS/cm]**	**Concentration [meq/L]**										**N**

					**H^+^**	**Na^+^**	**NH_4_^+^**	**K^+^**	**Mg^2+^**	**Ca^2+^**	**Cl^-^**	**NO^3-^**	**SO_4_^2-^**	**TIC**	
**Urban coastal**	A	max	7.20	222	1.15*10^-3^	0.92	0.37	0.27	0.45	1.40	1.08	0.28	1.40	5.16	6
		mean	6.57	171	4.10*10^-4^	0.52	0.23	0.16	0.23	0.84	0.62	0.14	0.65	3.32	
		min	5.94	117	6.31*10^-5^	0.26	0.085	0.075	0.064	0.36	0.26	0.076	0.32	2.17	

	Pc	max	6.98	747	6.03*10^-3^	0.63	0.42	0.375	0.39	1.81	1.00	0.48	1.39	4.95	20
		mean	6.46	195	6.68*10^-4^	0.19	0.27	0.12	0.13	0.81	0.51	0.12	0.48	2.63	
		min	5.22	63	1.05*10^-4^	0.024	0.063	0.024	0.022	0.24	0.14	0.032	0.14	0.83	

	Pm	max	7.35	482	1.62*10^-3^	1.07	1.18	0.43	0.77	2.49	1.71	0.72	1.94	8.24	38
		mean	6.49	142	4.92*10^-4^	0.28	0.25	0.12	0.18	0.75	0.53	0.13	0.49	2.69	
		min	5.79	29	4.51*10^-5^	0.013	0.029	0.0095	0.013	0.086	0.062	0.007	0.030	0.39	

**Urban inland**	A	max	7.99	287	3.55*10^-4^	0.28	0.70	0.22	0.29	1.42	0.48	0.49	1.50	3.98	6
		mean	7.05	199	1.55*10^-4^	0.19	0.38	0.12	0.14	0.76	0.33	0.23	0.70	2.94	
		min	6.45	31	1.02*10^-5^	0.092	0.21	0.056	0.066	0.24	0.13	0.042	0.25	1.46	

	Pc	max	8.02	532	7.08*10^-4^	0.68	0.67	0.26	0.62	2.80	0.97	0.81	4.46	10.1	41
		mean	6.99	257	1.68*10^-4^	0.19	0.35	0.10	0.17	1.16	0.26	0.19	1.25	3.73	
		min	6.15	68	9.55*10^-6^	0.0030	0.067	0.019	0.013	0.21	0.075	0.0042	0.32	1.20	

	Pm	max	7.23	265	2.14*10^-3^	0.43	0.59	0.36	0.22	1.27	0.40	0.51	2.50	5.22	37
		mean	6.44	123	4.97*10^-4^	0.10	0.31	0.07	0.05	0.42	0.15	0.09	0.59	1.81	
		min	5.67	29	5.91*10^-5^	0.020	0.111	0.025	0.003	0.037	0.027	0.001	0.11	0.52	

**Rural**	Pc	max	8.76	396	6.92*10^-2^	0.39	0.84	0.17	0.18	1.78	0.34	1.74	1.07	6.20	46
		mean	7.00	71.8	1.81*10^-3^	0.060	0.20	0.046	0.032	0.238	0.092	0.10	0.30	1.11	
		min	4.16	20	1.74*10^-6^	0.0087	0.043	0.010	0.00083	0.048	0.0085	0.013	0.065	0.27	

	Pm	max	7.73	276	5.01*10^-2^	0.96	0.46	0.11	0.11	0.45	0.20	0.33	1.22	2.73	40
		mean	6.64	68.4	2.05*10^-3^	0.08	0.19	0.050	0.026	0.17	0.090	0.066	0.31	1.01	
		min	4.30	19	1.86*10^-5^	0.0025	0.039	0.0067	0.00083	0.027	0.0037	0.016	0.030	0.28	

N = Number of samples

**Table 7.** Dew chemistry in relation to synoptic situation (AS-anticyclonic; TS-transitional; CS-cyclonic).

**Land use**	**Synoptic situations**		**pH**	**Conductivity [μS/cm]**	**Concentration [meq/l]**										**N**

					**H^+^**	**Na^+^**	**NH_4_^+^**	**K^+^**	**Mg^2+^**	**Ca^2+^**	**Cl^-^**	**NO^3-^**	**SO_4_^2-^**	**TIC**	
**Urban coastal**	**AS**	max	7.35	747	6.03*10^-3^	1.07	0.42	0.37	0.77	2.49	1.71	0.54	1.94	8.24	38
		mean	6.57	190	4.98*10^-4^	0.30	0.28	0.13	0.19	0.91	0.58	0.15	0.58	3.07	
		min	5.22	54.6	4.47*10^-5^	0.024	0.063	0.020	0.016	0.24	0.14	0.032	0.13	0.83	

	**TS**	max	6.96	334	1.26*10^-3^	0.97	0.45	0.43	0.43	1.71	1.65	0.72	1.19	6.41	13
		mean	6.56	152	3.81*10^-4^	0.30	0.19	0.14	0.18	0.81	0.57	0.15	0.53	2.88	
		min	5.90	31.6	1.10*10^-4^	0.024	0.087	0.0095	0.013	0.11	0.080	0.042	0.15	0.70	

	**CS**	max	6.75	236	1.62*10^-3^	0.65	1.18	0.25	0.29	0.72	1.13	0.096	0.60	4.81	13
		mean	6.17	85.0	8.21*10^-4^	0.18	0.26	0.089	0.10	0.34	0.35	0.051	0.26	1.57	
		min	5.79	28.8	1.78*10^-4^	0.013	0.029	0.016	0.026	0.086	0.062	0.0066	0.030	0.39	

**Urban inland**	**AS**	max	8.02	881	9.77*10^-4^	0.68	0.70	0.36	1.14	4.43	0.97	0.81	4.46	10.9	63
		mean	6.88	224	2.24*10^-4^	0.15	0.35	0.10	0.14	0.97	0.25	0.16	1.08	3.27	
		min	6.01	29.2	9.55*10^-6^	0.0030	0.067	0.019	0.0075	0.066	0.027	0.0042	0.19	0.78	

	**TS**	max	7.67	530	9.55*10^-4^	0.25	0.48	0.16	0.49	1.90	0.40	0.36	1.66	5.10	12
		mean	6.45	158	5.20*10^-4^	0.10	0.26	0.072	0.12	0.63	0.18	0.14	0.64	2.17	
		min	6.02	32.1	2.14*10^-5^	0.020	0.10	0.025	0.0025	0.037	0.047	0.0010	0.11	0.52	

	**CS**	max	7.23	184	2.14*10^-3^	0.17	0.54	0.092	0.14	1.11	0.39	0.35	1.49	3.39	10
		mean	6.45	112	5.90*10^-4^	0.083	0.29	0.055	0.045	0.38	0.15	0.10	0.53	1.66	
		min	5.67	42.5	5.89*10^-5^	0.032	0.15	0.026	0.0050	0.046	0.034	0.0021	0.13	0.62	

**Rural**	**AS**	max	8.76	396	6.92*10^-2^	0.39	0.84	0.17	0.18	1.78	0.34	1.74	1.07	6.20	67
		mean	6.93	66.7	1.46*10^-3^	0.061	0.18	0.047	0.030	0.21	0.090	0.087	0.29	1.04	
		min	4.16	19.3	1.74*10^-6^	0.0078	0.039	0.010	0.00083	0.028	0.0085	0.013	0.030	0.27	

	**TS**	max	7.47	182	5.01*10^-2^	0.96	0.33	0.11	0.11	0.37	0.20	0.22	1.22	2.73	12
		mean	6.48	62.7	5.21*10^-3^	0.12	0.17	0.052	0.034	0.14	0.067	0.050	0.35	1.02	
		min	4.30	22.1	3.39*10^-5^	0.0052	0.086	0.0067	0.0083	0.031	0.0037	0.016	0.084	0.37	

	**CS**	max	7.39	276	2.40*10^-3^	0.12	0.46	0.093	0.043	0.37	0.20	0.33	0.61	1.92	7
		mean	6.55	117	6.37*10^-4^	0.057	0.30	0.057	0.023	0.23	0.14	0.11	0.37	1.33	
		min	5.62	39.2	4.07*10^-5^	0.033	0.22	0.018	0.0083	0.081	0.065	0.030	0.15	0.88	

N = Number of samples
